#  Chondrosarcoma mimicking MRI of the osteonecrosis of the femoral head: a case report

**DOI:** 10.1259/bjrcr.20170098

**Published:** 2018-03-08

**Authors:** Junya Shimizu, Makoto Emori, Satoshi Nagoya, Mikito Sasaki, Kenji Tateda, Toshihiko Yamashita

**Affiliations:** 1 Department of Orthopaedic Surgery, Sapporo Medical University School of Medicine, Sapporo, Japan; 2 Department of Musculoskeletal Biomechanics and Surgical Development, Sapporo Medical University, Sapporo, Japan

## Abstract

A 25-year-old female visited our hospital with an 8-year history of arthralgia in the right hip joint. Plain radiography of the hip revealed a well-demarcated radiolucent lesion with a thin sclerotic rim in the epiphysis of the femoral head. *T*_1_ weighted MRI revealed the demarcation line of a low-signal-intensity band in the femoral head. We were aware that this band did not split the signal of adipose tissue in the bone marrow. In cases of osteonecrosis, we usually find a low-signal-intensity band splitting the signal of normal bone marrow. However, we could not see such a low-signal-intensity band in this case. Therefore, we decided to perform other studies. Contrast-enhanced *T*_1_ weighted MRI showed remarkable enhancement in the segment proximal to the low-signal-intensity band, indicating that this lesion might have blood perfusion. We decided to perform a bone biopsy to clarify the diagnosis. Histopathological examination of the biopsy specimen revealed chondrosarcoma. We found that contrast-enhanced MRI plays an important role to rule out osteonecrosis of the femoral head.

## Clinical Presentation

### Background

Non-traumatic osteonecrosis of the femoral head (ONFH) is one of the most common hip diseases between adolescence and midlife.^1^ Alcohol abuse and corticosteroid use have been identified as risk factors for ONFH.^2^ Although early-stage radiography displays normal or well-marginated sclerotic findings in the proximal end of the femoral head, a low-signal-intensity band on *T*_1_ weighted MRI is usually pathognomonic for ONFH.^3, 4^ As such, radiologists and orthopaedic surgeons tend to interpret this characteristic finding as consistent with ONFH. However, there are several reports of misdiagnosis of ONFH as other diseases, such as transient osteoporosis of the hip and subchondral bone insufficiency fracture.^5^ To our knowledge, there are few reports of malignant bone tumours mimicking ONFH. Herein, we describe the MRI findings of chondrosarcoma in the femoral head mimicking those of ONFH in a 25-year-old female.

## Case Report

A 25-year-old female presented to our orthopaedic hospital with an 8 year history of arthralgia in the right hip. Under an initial diagnosis of ONFH, she had visited the Department of Orthopedic Surgery of Sapporo Medical University Hospital. She had no history of trauma, corticosteroid use, alcohol abuse, or other condition related to osteonecrosis. On admission, her range of hip motion was slightly restricted and other physical examinations were nonspecific.

## Investigations and Imaging Findings

**Figure 1.**
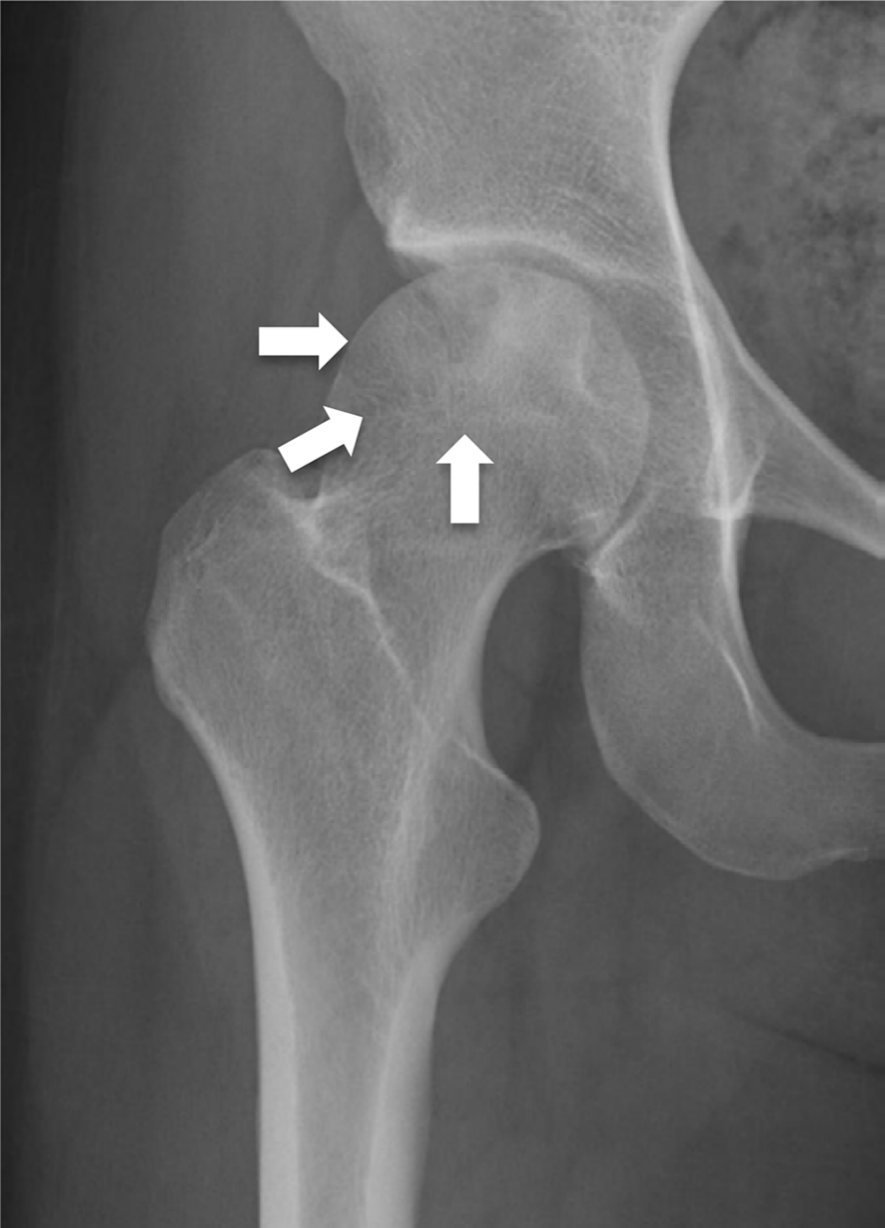
Radiograph showing an epiphyseal osteolytic lesion with a sclerotic band in the right femur (arrow).

**Figure 2.**
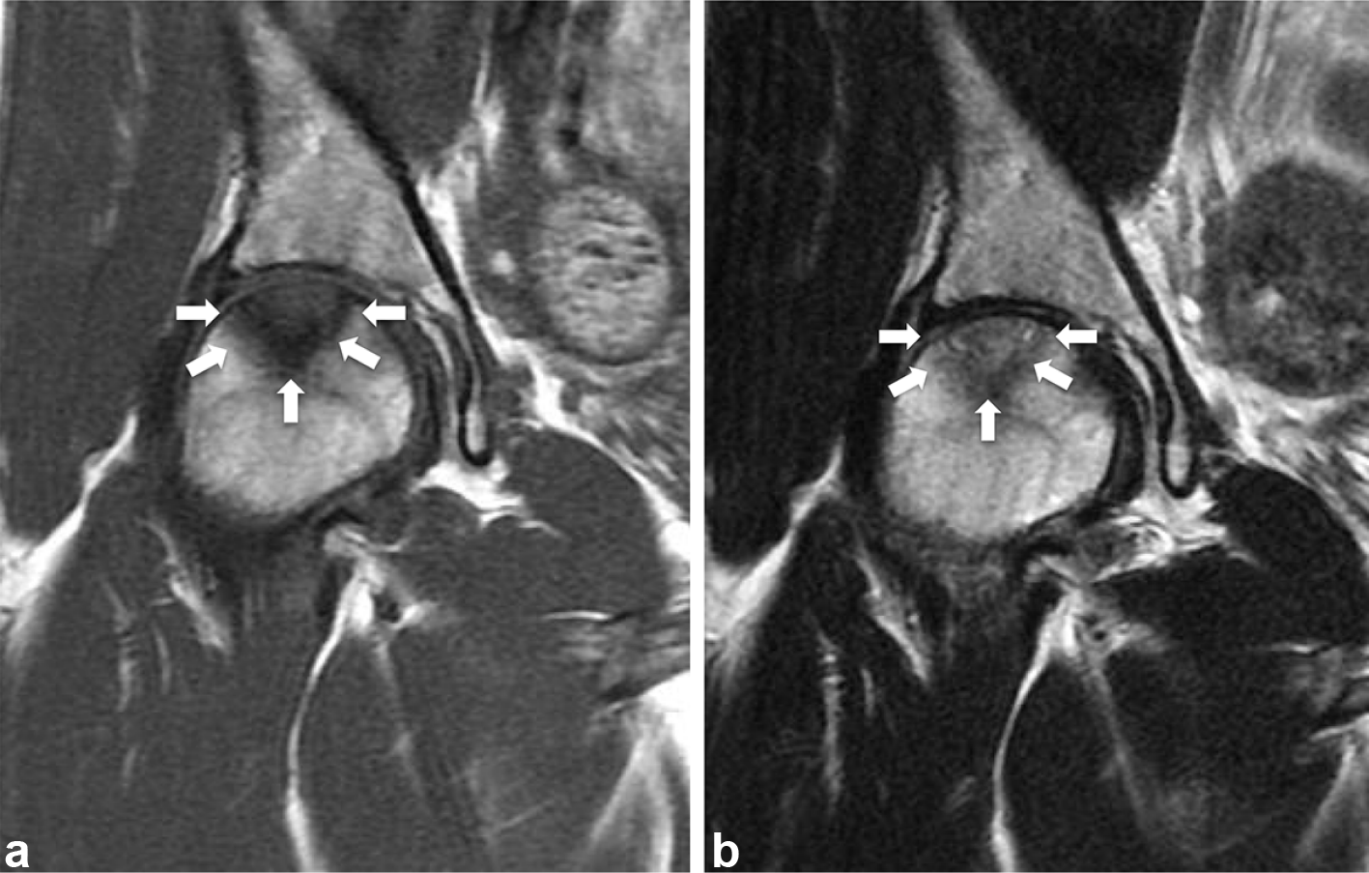
(a) Coronal *T*_1_ weighted MRI showing a low-signal-intensity band (arrow). (b) Coronal *T*_2_ weighted MRI showing an iso-/high-signal intensity mass proximal to the low-signal-intensity band (arrow).

**Figure 3.**
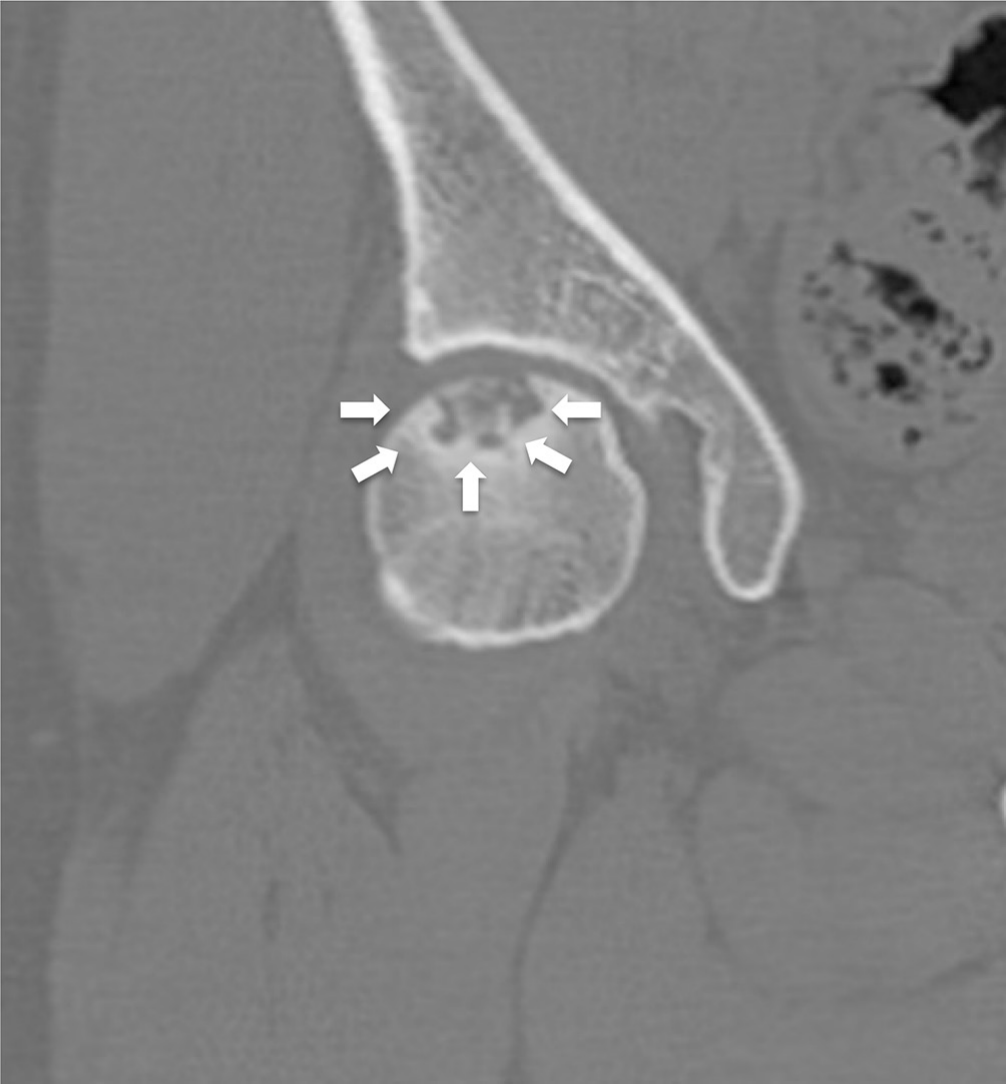
Coronal CT scan showing a well-defined bony lytic lesion with calcification in the epiphysis of the left femur (arrow).

Laboratory data were all within normal range. Plain radiography of the hip revealed a well-demarcated radiolucent lesion with a thin sclerotic rim in the epiphysis of the femoral head (Figure 1). We initially suspected ONFH and performed MRI. *T*_1_ weighted MRI revealed the demarcation line of a low-signal-intensity band in the weight-bearing portion of the femoral head (Figure 2a). *T*_2_ weighted MRI showed a high-signal-intensity lesion proximal to the band (Figure 2a). CT showed a well-defined low-density area demarcated by a sclerotic rim, corresponding to the weight-bearing lesion observed on MRI (Figure 3) and displayed segmental collapse with residual trabecular bone matrix in the lesion. Based on these findings, the radiologist diagnosed ONFH.

**Figure 4.**
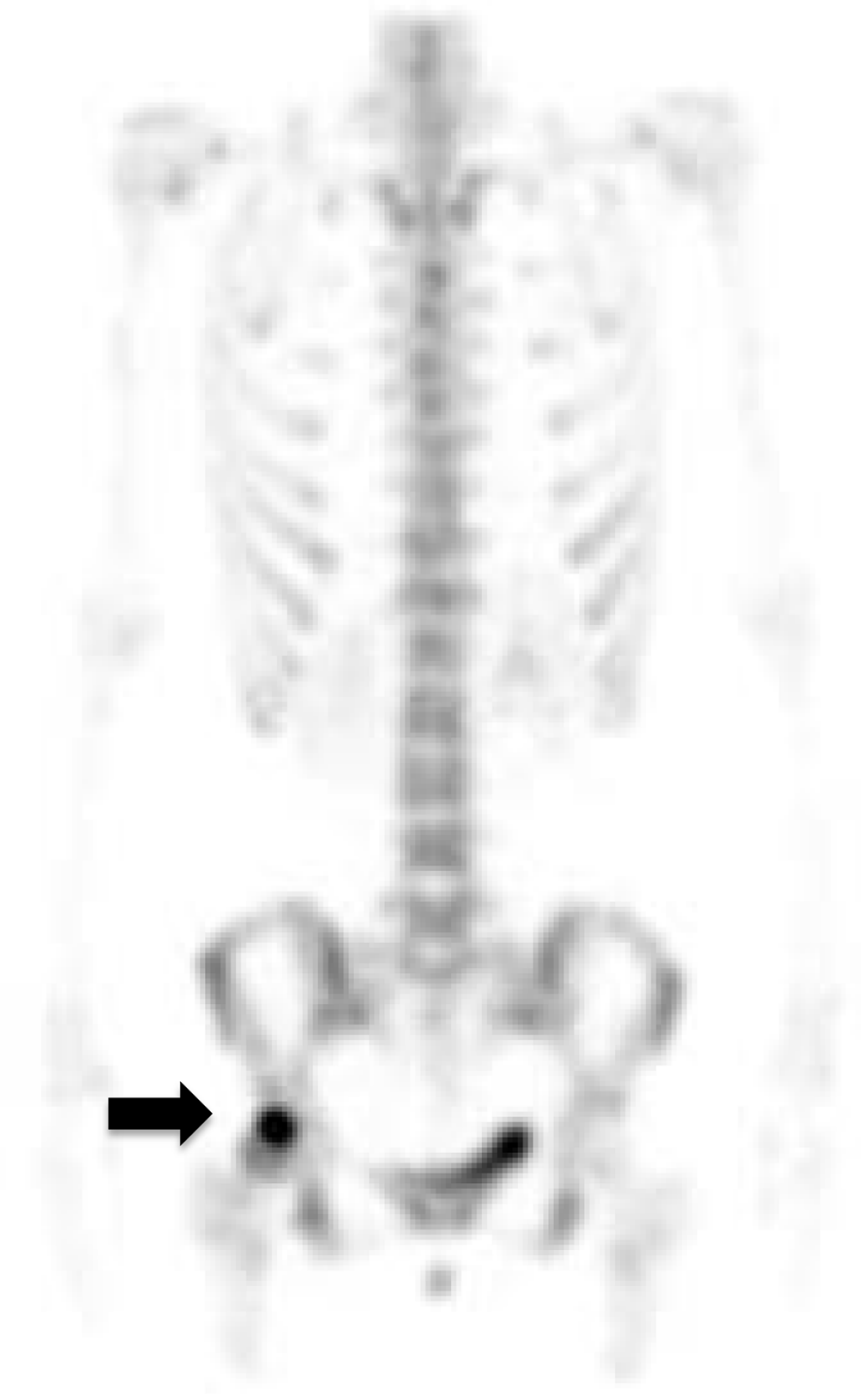
Bone scintigraphy showing increased focal uptake in the lesion (arrow).

**Figure 5.**
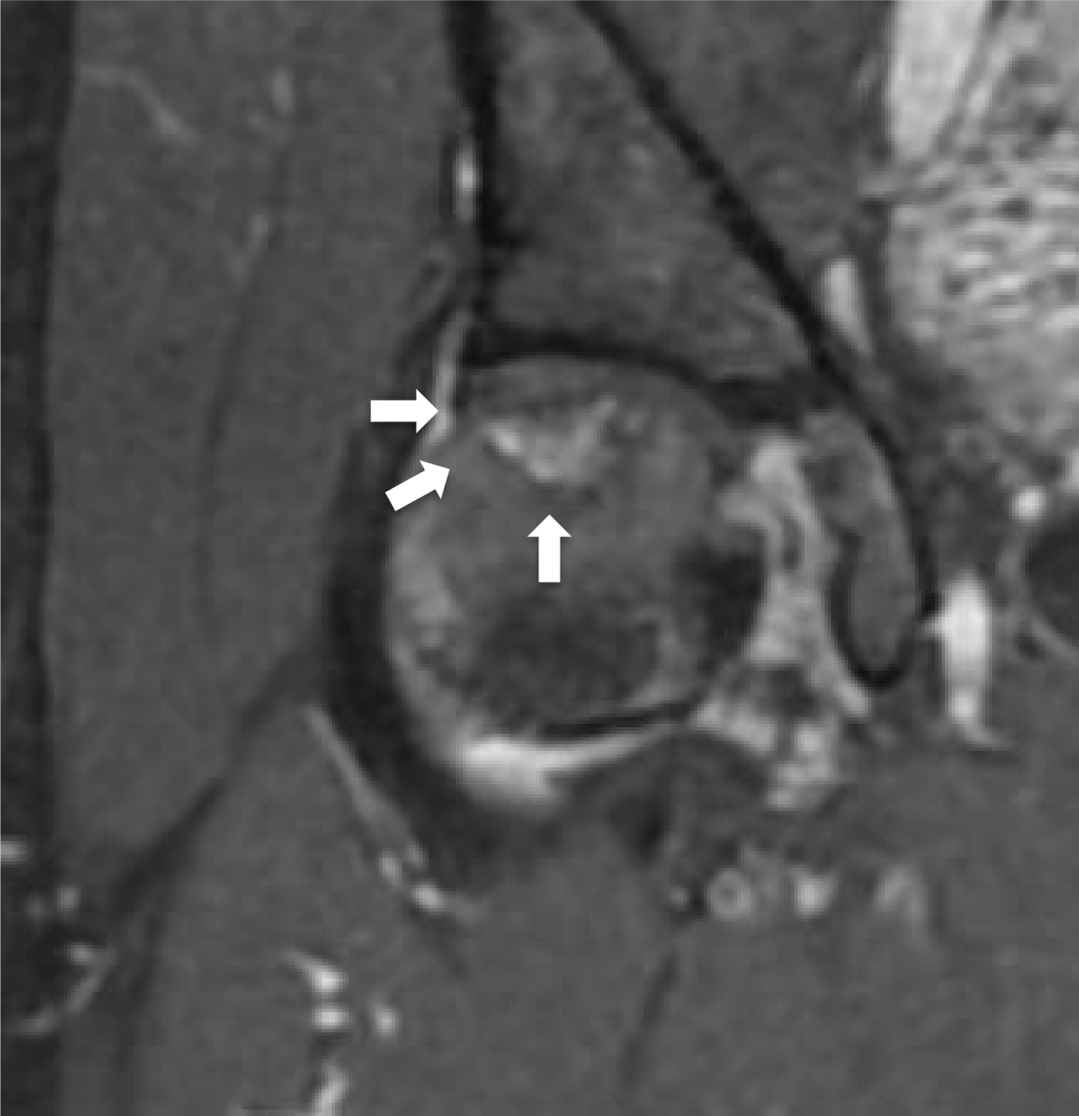
Contrast-enhanced *T*_1_ weighted MRI showing enhancement proximal to the low-signal intensity band.

However, prior to performing anterior transtrochanteric osteotomy, because we were aware that the low-signal-intensity band did not split the signal of adipose tissue in bone marrow. So, we decided to perform other studies to exclude osteonecrosis.^6, 7^ Bone scintigraphy demonstrated increased focal uptake in the lesion (Figure 4), while contrast-enhanced *T*_1_ weighted MRI showed remarkable enhancement in the segment proximal to the low-signal-intensity band, indicating that the lesion might have blood perfusion (Figure 5). Furthermore, enhanced lesions were found in the other intra-articular regions. We did not perform diffusion MRI at that time. In consideration of these findings excluding ONFH as a definitive diagnosis, we decided to perform a bone biopsy using an anterolateral approach. Because needle biopsy appears to be an unreliable method to reach the small lesions in the femoral head, even CT-guided procedure was used. Since the collected materials might be too small to confirm an appropriate diagnosis, we decided to perform an open biopsy. Histopathological findings of the biopsy specimen that revealed that the less cellular part had only a few double nucleated cells and moderate atypia and lobulated architecture, with abundant cartilaginous matrix separated by narrow fibrovascular bands, indicating conventional chondrosarcoma.

## Treatment and Outcome

**Figure 6.**
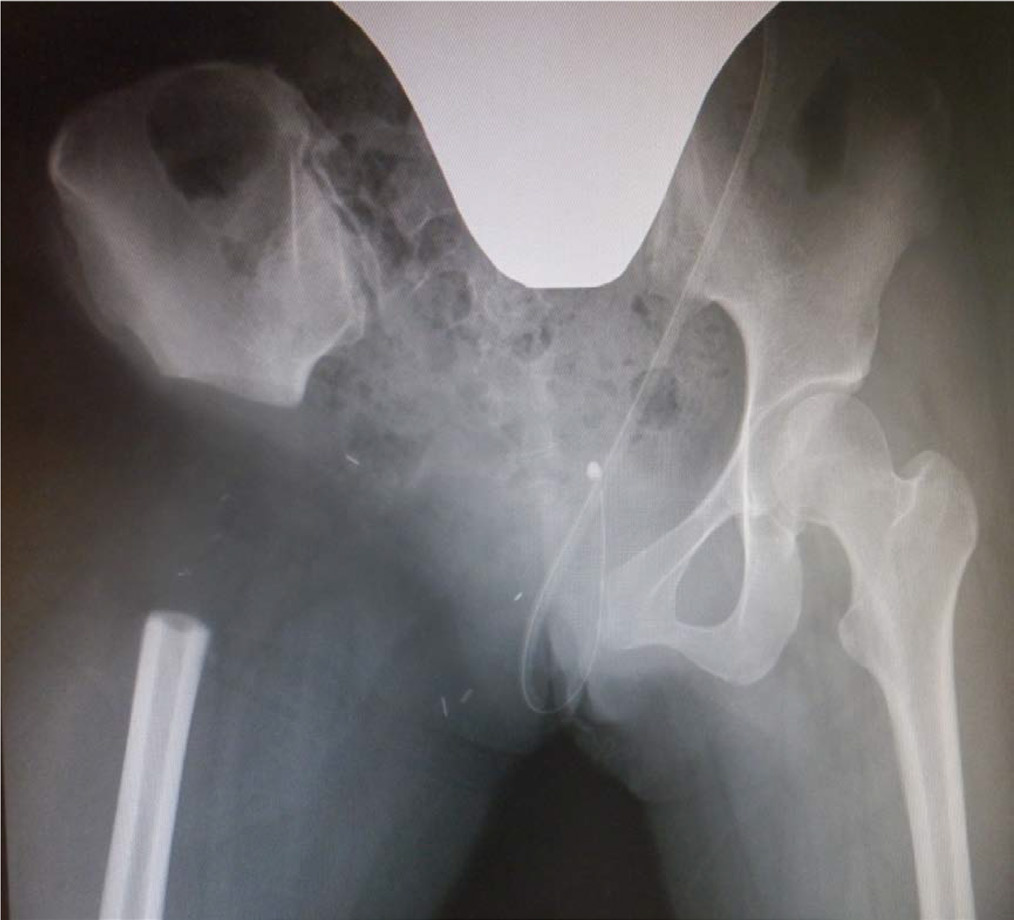
Radiograph of the pelvis after wide tumour resection at the wing of the ilium, pubic symphysis, and femur under the lesser trochanter.

**Figure 7.**
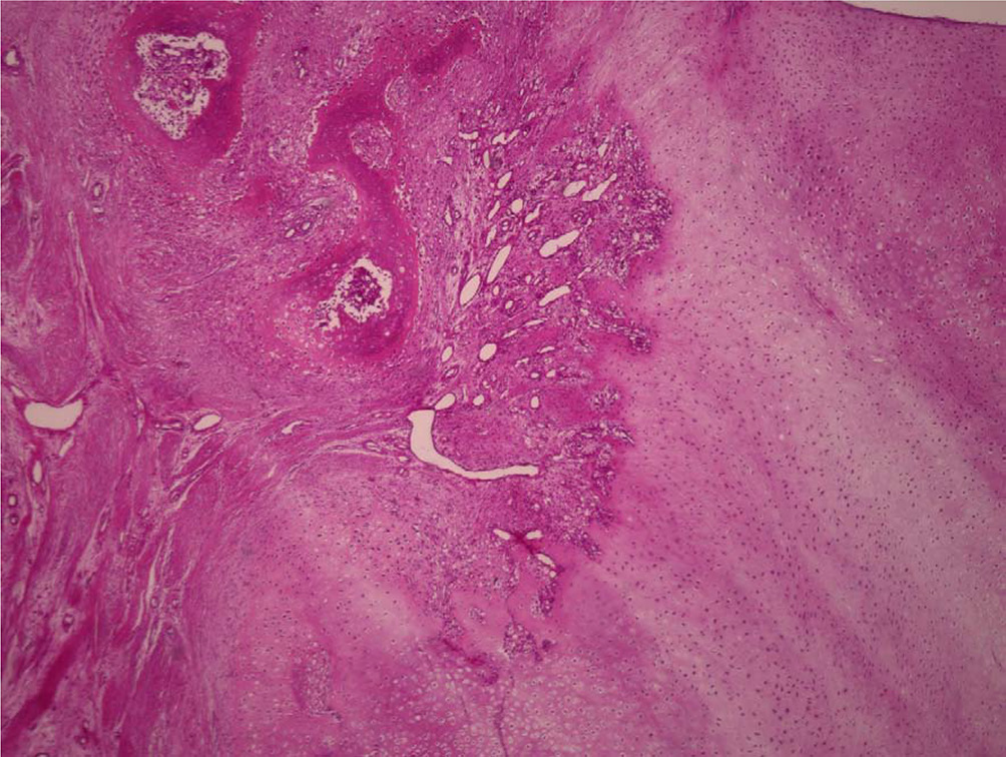
Low-power microscopic image showing a multiloculated tumour in a chondroid background under subchondral bone (haematoxylin and eosin staining, magnification × 100). The less cellular part has only a few double-nucleated cells, moderate atypia, and lobulated architecture, with abundant cartilaginous matrix separated by narrow fibrovascular bands.

We performed wide tumour resection surgery with Type II + III resection and internal hemipelvectomy for limb salvage, because intra-articular spread of the tumour was evident on enhanced MRI prior to open biopsy (Figure 6).^8^ The final pathological diagnosis of the resected tumour was conventional chondrosarcoma (Figure 7). 5 years after surgery, there was no recurrence or metastasis.

## Discussion

A low-signal-intensity band in the femoral head on *T*_1_ weighted MRI is usually pathognomonic for ONFH. In this patient, however, the low-signal-intensity band did not split the signal of adipose tissue in bone marrow, causing us to doubt the diagnosis of ONFH. Further imaging studies and open biopsy revealed that this lesion was actually conventional chondrosarcoma arising from the epiphysis of the proximal end of the femur.

The critical findings of ONFH include not only presence of a low-signal-intensity band in the femoral head, but also the low-signal intensity band splitting the signal of adipose tissue in bone marrow, in other words, a three-layered structure on *T*_1_ weighted MRI.^4^ In absence of this three-layered structure, contrast-enhanced MRI must be employed to investigate whether blood perfusion exists in the lesion, because the area proximal to the low-signal-intensity band might not show enhancement in patients with ONFH. The three-layered structure (low-, high-, and low-signal) on *T*_1_ weighted MRI reflects the differences in necrotic, vascularized granulation tissue, and normal areas in the femoral head.

Conventional chondrosarcoma accounts for approximately 20% of malignant bone tumours. The majority of patients are over 50 years of age; however, this patient was only 25 years old, much younger than the majority of patients with chondrosarcoma. It has been reported that 84% of chondrosarcomas occur in the trunk and upper end of the femur and humerus.^9^ Almost 90% of all chondrosarcomas of the long bones arise in the metaphysis or diaphysis; tumours in the epiphysis are extremely rare.^9^ To our knowledge, although a few cases of mesenchymal chondrosarcoma in the proximal femur have been reported, there has been no case of conventional chondrosarcoma arising in the epiphysis of the proximal femur.

When comparing the present case with ONFH, chondrosarcoma and osteonecrosis may be different in terms of precise signal intensity on *T*_1_ weighted MRI. In this case of chondrosarcoma, although a low-signal-intensity band typically suggesting ONFH was observed, the lesion proximal to this band had iso-/high-signal intensity, and not quite the low-signal-intensity attributable to ONFH, which demonstrates a three-layered structure on *T*_1_ weighted MRI. Accurate diagnosis of ONFH requires this three-layered structure, in which the low-signal-intensity band splits the signal of adipose tissue in bone marrow. In addition, contrast-enhanced MRI is very useful to distinguish a tumour lesion from a necrotic lesion.^6^ The segment proximal to the low-signal-intensity band shows enhancement in the case of a tumour lesion, but is not enhanced in the case of a necrotic lesion. In the present case, *T*_2_ weighted MRI failed to display quite high-signal-intensity that was consistent with typical chondral matrix in conventional chondrosarcoma.

Contrast-enhanced MRI plays an important role to rule out ONFH. When we encounter a low-signal-intensity band without the three-layered structure on *T*_1_ weighted MRI, we should perform additional studies, including contrast-enhanced MRI, with consideration of a tumour-related lesion, such as chondrosarcoma.

## Learning points

1. Conventional chondrosarcoma in the epiphysis of the long bones is very rare.
2. In general, the MRI of the ONFH demonstrates a three-layered structure on *T*_1_ weighted MRI splitting the signal of the normal bone marrow. In the absence of a three-layered structure, we should perform contrast-enhanced.
3. It is difficult to make an exact diagnosis from the imaging alone. Pathological imaging correlation is very necessary for the exact diagnosis and for differentiating between the benign and the malignant forms of the disease.
